# Environmental Arsenic Exposure, Biomarkers and Lung Function in Children from Yaqui Communities in Sonora, Mexico

**DOI:** 10.3390/jox15040115

**Published:** 2025-07-08

**Authors:** Ana G. Dévora-Figueroa, Anaid Estrada-Vargas, Jefferey L. Burgess, Paloma I. Beamer, José M. Guillen-Rodríguez, Leticia García-Rico, Diana Evelyn Villa-Guillen, Iram Mondaca-Fernández, Maria M. Meza-Montenegro

**Affiliations:** 1 Programa de Doctorado en Ciencias Especialidad en Biotecnología, Instituto Tecnológico de Sonora, 5 de febrero 818 Sur, Ciudad Obregón 85000, Sonora, Mexico; ana.devora114123@potros.itson.edu.mx (A.G.D.-F.); anaid.estrada124137@potros.itson.edu.mx (A.E.-V.); 2 Community, Environment & Policy Department, Mel & Enid Zuckerman College of Public Health, University of Arizona, Tucson, AZ 85721, USA; jburgess@arizona.edu (J.L.B.);; 3 Arizona Cancer Center, College of Medicine, University of Arizona, Tucson, AZ 85724, USA; 4 Centro de Investigación en Alimentación y Desarrollo, A.C., Carretera Gustavo Astiazarán 46, Hermosillo 83304, Sonora, Mexico; lgarciar@ciad.mx; 5 Hospital General del Estado de Sonora, Blvd. Luis Encinas J. S/N, San Benito, Hermosillo 83000, Sonora, Mexico; 6 Departamento de Recursos Naturales, Instituto Tecnológico de Sonora, 5 de febrero 818 Sur, Ciudad Obregón 85000, Sonora, Mexico; hmondaca@itson.edu.mx

**Keywords:** arsenic exposure, biomarkers, Yaqui children, lung function

## Abstract

Arsenic exposure in children and adults has been associated with respiratory symptoms, respiratory infections, and decreased lung function. The goal of this study was to evaluate the relationship between environmental arsenic exposure and serum pneumoproteins and lung function. A cross-sectional study was conducted including 175 children exposed to arsenic by drinking water (range: 7.4 to 91 µg/L) and soil (range: 4.76 to 35.93 mg/kg), from some Yaqui villages. Arsenic was analyzed in dust and urine using field-portable X-ray fluorescence spectrometry and ICP/OES, respectively. Serum was analyzed for Clara Cell protein (CC16) and Matrix Metalloproteinase-9 (MMP-9) using immunoassays, and lung function was evaluated by spirometry. The results showed that increased arsenic in drinking water was associated with reduced forced expiratory volume in one second (FEV_1_)/forced vital capacity (FVC) ratio (β = −0.027, *p* = 0.0000) whereas, contrary to expectations, arsenic in dust was associated with increased FEV_1_/FVC (β = 0.004, *p* = 0.0076). Increased urinary arsenic was associated with reduced % predicted FEV_1_ (β = −0.723, *p* = 0.0152) and reduced FEV_1_/FVC ratio (β = −0.022, *p* = 0.0222). Increased serum MMP-9 was associated with reduced FEV_1_/FVC ratio (β = −0.017, *p* = 0.0167). Children with % predicted FEV_1_ values less than 80 had the lowest levels of CC16 (Median 29.0 ng/mL, IQR 21.3, 37.4, *p* = 0.0148). As a conclusion, our study evidenced an impairment in lung function in children exposed to low arsenic levels.

## 1. Introduction

Environmental contamination by arsenic has become a worldwide concern. Arsenic is the twentieth most common element in the earth’s crust [1], so it is naturally present in the environment. In addition, arsenic has been used for centuries in agriculture, pharmaceuticals, mining, etc., and this metalloid is commonly found as a contaminant in environmental media such as drinking water, food, soil, and dust. It is estimated that up to 220 million people are at risk of elevated arsenic concentrations in their groundwater [2]. Moreover, dust has become a key source of arsenic exposure in arid zones [3,4].

Arsenic inhalation of suspended contaminated particles and arsenic ingestion from soil are sources of exposure which have not been extensively studied [3,5,6,7,8], even though arsenic in surface soils can easily be suspended in blowing dust and transported over long distances [9]. Soil dust represents small particles (<100 μm) that have settled onto surfaces due to wet or dry deposition and is a major carrier of metal contaminants that can accumulate over a long period of time [10,11,12]. The fine grain (<20 µm) dust fraction can most easily be resuspended by wind or emitted by anthropogenic activities and represents an important fraction of potentially inhaled and ingested dust [4,13]. In arid and semi-arid regions, like southern Sonora, Mexico, respirable dust levels are potentially problematic, due to the increased windborne transport of metal-laden dust particles, especially because Sonora state has natural arsenic occurrence in the ground water and soils [2,3,5,14]. 

Arsenic is classified as a Group 1 lung carcinogen by the International Agency of Research on Cancer [15]. Lung tissue is one of the most important target organs of arsenic. It has also been reported that exposure to arsenic via drinking water in utero and during early childhood is associated with impaired lung function, as a chronic inflammation response to the metalloid [16,17,18,19]. Some studies have associated arsenic via drinking water ingestion with pneumonia, allergies, cough, asthma, bronchitis, chronic obstructive pulmonary disease (COPD), respiratory disease mortality, increased respiratory symptoms during childhood [20,21,22], and decreased lung function with altered spirometric indices of FEV_1_ and FVC [17,18,21].

Biomarkers have been used to measure the effects of exposure to various environmental media in an effort to improve human health risk assessment [5,23,24]. Clara cell protein (CC16) is a biomarker of lung epithelium integrity and appears to be a key protecting protein which is reduced in response to inhaled toxicants [3,23,25]. Another pulmonary biomarker is matrix metalloproteinase-9 (MMP-9), which has been found to increase with low-level arsenic exposure in humans [26]. Previous studies reported serum levels of these two pneumoproteins (CC16 and MMP-9) associated with arsenic exposure through drinking water in children and adults from Yaqui villages in southern Sonora, but there are no previous reports in these communities relating arsenic exposure via dust and biomarkers of lung inflammation, with spirometric indices for lung function in this same population, even though the dust resuspension in these villages is a potentially serious respiratory health problem [22,27]. 

Spirometry is one of the most readily available and useful tests to determine spirometric indices for lung function; as such, it uses FVC, FEV_1_, and the FEV_1/_ FVC ratio to diagnose respiratory abnormalities and to quantify the severity of respiratory impairment [28]. Respiratory alterations can be identified from patterns of lung function such as restrictive and obstructive airflow [17]. There are scarce reports in the literature which have been associated with arsenic exposure by drinking water and lung function parameters in children and adults, showing, in most cases, that arsenic was associated with restrictive impairments [17,18,19]. Human and mouse models have shown that in utero and early life exposures to arsenic can result in alterations in adult lung function and lung disease [19]. However, at present, no studies exist regarding the relationship of arsenic exposure by various environmental media, inflammation biomarkers, and lung function in children. In this context, the main goals of this study were as follows: (a) Evaluate the relationship between arsenic exposure by drinking water, urban dust, and urinary arsenic and spirometric indices in children and (b) Evaluate the association between serum MMP-9 and CC16 levels and spirometric indices. 

## 2. Materials and Methods

### 2.1. Study Population, Recruitment and Sample Selection

The study protocol, questionnaires and informed consent were approved by The Ethics Committee of the Instituto Tecnológico de Sonora (24 February 2015), according the nature of the project and the subjects’ benefits and rights conforming to Helsinki Declaration. The children included in this study are a subgroup of those reported in an earlier cross-sectional study carried out from 2015 to 2016 with healthy children (*n* = 216) aged between 6 and 16 years old. Children were selected at random through meetings with the directors and academic personnel from the elementary schools and door-to-door contact in each of the three Yaqui villages (Pótam, Vícam and Cócorit). The inclusion criteria required them having been exposed their entire life to arsenic by drinking water and dust, including in utero exposure [22]. These villages do not have arsenic point sources such as mining or smelting operations near the vicinity of sample collections.

### 2.2. Interviews

The participants were informed of the objective and methodology of the investigation. All parents gave verbal approval and signed the consent forms for their children before their participation in the study. A questionnaire was used to obtain information from the children and their parents regarding residential history, diet, number of smokers in the home, respiratory diseases, and respiratory symptoms. The privacy of the personal data of each child was carefully protected.

### 2.3. Environmental Sample Collection

#### Soil Samples

**Figure 1:**
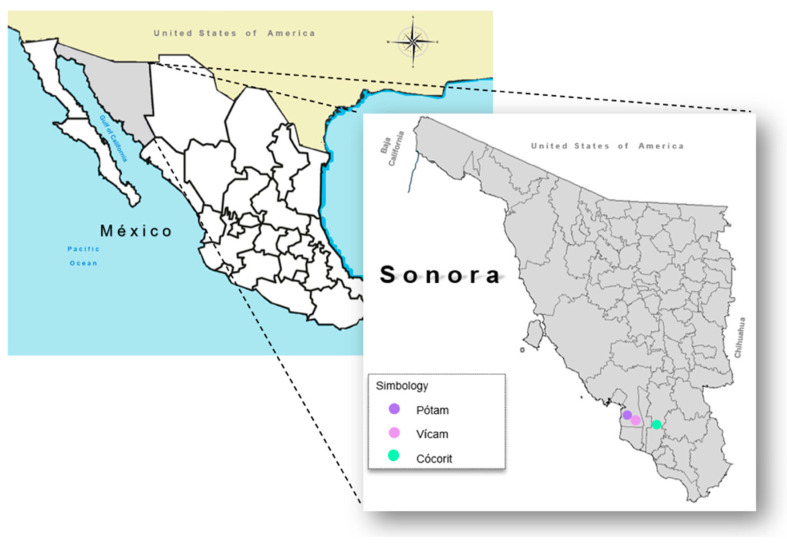
Location of studied Yaqui communities in south Sonora, México.

Soil samples (*n* = 197) were obtained from unpaved roads and a few from paved streets in Pótam (*n* = 56), Vícam (*n* = 78), and Cócorit (*n* = 63) [Figure 1]. These samples were taken where children spent more time playing and where there was some vehicular traffic. Sampling was carried out according to the methodology established by the Mexican norm “Soil sampling for identification and quantification of metals and metalloids, and sample management” [29]. The sampling was exploratory in the urban areas, i.e., obtaining representative and random soil samples close to the participant’s households to establish the presence of arsenic contamination. Soil sampling was carried out in two ways due to the nature of the samples: (1) Soil samples from unpaved roads were collected using a stainless-steel gardener shovel. The samples were collected by taking 5 samples of the surface soil (depth: 0 to 5 cm) and mixing to obtain a composite sample. (2) Samples from paved streets were collected by sweeping a section of the street using a broom and plastic collector. The collected samples were placed in one-gallon polyethylene Ziploc bags previously labeled with location information. The collected soil samples (approximately 500 g) were dried at room temperature and sieved through a 635-mesh screen, obtaining the dust samples with particle sizes between 20 and <20 µm.

### 2.4. Drinking Water Samples

Each village’s drinking water wells were sampled monthly for a year, and an average arsenic total was calculated for each of these communities. Water samples were obtained directly from the water pump heads and stored in polypropylene bottles. The samples were kept at −20 °C until the analysis of total arsenic was performed [30].

### 2.5. Biological Sampling

#### Urine Collection

The first morning void was collected from each participant in 100 mL polypropylene sterilized containers. Once the samples were obtained, they were transported in coolers at an approximate temperature of 4 °C, until they arrived at the Toxicology and Public Health Laboratory where they were stored at −40 °C until their analysis.

### 2.6. Blood Collection

For each child, blood samples were obtained by venipuncture, using 5 mL BD vacutainer tubes for blood serum. Then the serum was separated by centrifugation (1438× *g* for 12 min) and kept frozen at −40 °C until the analysis.

### 2.7. Determination of Total Arsenic

#### Dust Samples

Triplicate dust samples were analyzed for total arsenic using a portable X-ray Fluorescence (PXRF) Analyzer model Niton FXL GOLDD (Thermo Scientific, Inc., Waltham, MA, USA), following the method 6200 of the US Environmental Protection Agency [31]. To ensure the quality of the data, reference material SRM 2710a, Montana I Soil-Highly Elevated Trace Element Concentrations (NIST, Gaithersburg, MD, USA) was measured every ten samples, and the average recoveries of the study elements were 110%, with a coefficient of variation (CV) of 9.7% and a detection limit of 2 mg·kg^−1^.

### 2.8. Drinking Water

Analysis of total arsenic in water was undertaken as previously described by Vega-Millán et al. [22].

### 2.9. Urine

Urine samples were processed to quantify total arsenic according to the methodology reported by Vega-Millán et al. [22]. A portion of the urine (4 mL) was transferred to digestion vessels combined with 4 mL of concentrated nitric acid (HNO_3_ 67–70% J.T. Baker, Radnor, PA, USA) and 2 mL of hydrogen peroxide (H_2_O_2_ 30% J.T. Baker, Radnor, PA, USA) Samples were pre-digested at room temperature for 30 min and then placed in a MARS 6, Microwave Digestion System (CEM, Matthews, NC, USA) at 200 °C for 35 min. The resulting solutions were transferred to 25 mL volumetric flasks and filled with HPLC grade water. The detection of arsenic was carried out by Inductive Coupling Plasma with Optical Emission Spectroscopy (ICP/OES) model iCAP 7600 Duo (Thermo Scientific, Inc., Waltham, MA, USA). The 189.04 nm (axial) line was used, and the limit of quantification was 7 μg/L of arsenic. A certified reference material SRM 3669 “Arsenic Species in Frozen Human Material Urine” (elevated Levels), was used for quality control (NIST, Gaithersburg, MD, USA) with a recovery of 97% and a % CV of 1.3.

### 2.10. Determination of Lung Biomarkers Levels in Serum

The serum concentrations of CC16 and MMP-9 were measured as peripheral lung biomarkers by sandwich enzyme-linked immunosorbent assays (R&D Systems, Minneapolis, MN, USA, catalog numbers DUGB00 and DMP900, respectively). Samples were read to 450 nm with a background correction of 540 to 570 nm using a UV-VIS Microplate Spectrophotometer model Multiskan GO (Thermo Scientific, Inc., Waltham, MA, USA. The minimum detectable levels were 0.13 ng/mL for CC16 and 0.78 ng/mL for MMP-9.

### 2.11. Spirometry Tests

Lung function was determined in accordance with the American Thoracic Society guidelines [32], using an EasyOne spirometer in diagnostic mode for pulmonary function tests. The instrument was calibrated each morning prior to data collection with a 3 L syringe (Sensor Medics, New Berlin, WI, USA). Exclusion criteria for spirometry included a positive answer for any of the following questions in the last three months: thoracic or abdominal surgery, heart problem, eye surgery, admission to the hospital for any cardiac condition, and pregnancy among the girls was also exclusionary for spirometry. The procedure was explained in detail to the study participants who were allowed to practice with the equipment until they felt comfortable. Participants were instructed to take as deep a breath as possible and then blow as hard and long as possible into the spirometer using a nose clip and a disposable mouthpiece. Following a demonstration and practice with the mouthpiece, participants performed tests in a sitting position with active coaching. The maneuver was repeated until the spirometer indicated that satisfactory results were achieved (e.g., forced expiratory volume in one second (FEV_1_) and forced vital capacity (FVC) within 200 mL of previous values or the participant chose to stop). Each subject’s best trial (largest sum of FEV_1_ and FVC) was included in analyses. Spirometry data were reviewed by pulmonologists. The age, height, weight, gender, and race % predicted values for FEV_1_, FVC, and their ratio (FEV_1_/FVC) were recorded, using predicted values for Mexican–Americans in NHANES III [33]. Predicted percentages of ≥ 80% for FVC and FEV_1_ and a FEV_1_/FVC ratio of ≥0.7 were considered as cut off values for lung function tests to be normal, as generally used internationally [34]. Obstructive lung function was defined as having FEV_1_ < 80% and FEV_1_/FVC < 70% and restrictive lung function was defined as having FEV_1_ < 80% and FEV_1_/FVC > 70% [35].

### 2.12. Statistical Analysis

Descriptive statistics were used to calculate means and standard deviation for arsenic levels, age, gender, BMI, weight, height, schooling, and residency. ANOVA with Bonferroni correction was used to evaluate differences among children from the villages studied, for anthropometric and socio-demographic characteristics, lifestyle, arsenic levels in drinking water, dust and urine, and serum CC16 and MMP-9 levels. To determine the relationship between urinary arsenic, and biomarkers levels with the spirometric indices, a Pearson correlation was determined. To evaluate differences between spirometric indices, urinary arsenic, and CC16 and MMP-9 by village, a Mann–Whitney test was used with Bonferroni correction. Multiple linear regression analysis was used to assess the association between urinary arsenic, CC16, MMP-9, and spirometric indices, and in all cases the models were adjusted for the most common confounders previously reported in the literature (age, sex, BMI, and passive smoking). To evaluate multi-collinearity, the variance inflation factor (vif) was calculated for the final adjusted models. A vif > 5 for any variables may indicate cause for trouble. In the overall regression models, the interaction term between child’s sex, biomarkers levels, and spirometric indices values did not reach statistical significance with a range of *p*-values greater than 0.05. All statistical tests were performed using the alpha levels set at 0.05. Data were analyzed using Stata 17.0 software (College Station, TX, USA, 2021).

## 3. Results

### 3.1. Anthropometric Characteristics

The main characteristics of the study group are shown in Table 1. The study included girls (54.29%) and boys (45.71%) with a mean age of 9.77 ± 2.31 years for the total sample. Mean height was 1.42 ± 0.15 m, mean weight was 39.12 ± 14.87 kg, and the average BMI was 18.82 ± 4.36 kg/m^2^ (Table 1). None of these variables showed statistical differences by village (*p* > 0.05). Children from Cócorit had the highest percentage of passive smoking (43.64%), significantly greater than children from Pótam and Vícam with percentages of 21.74% and 9.80%, respectively.

### 3.2. Environmental Arsenic

Arsenic in urban dust for the three villages ranged from 4.76 to 35.93 mg/kg. The highest concentrations were found in Cócorit with a mean value of 20.25 ± 6.74 mg/kg, which was significantly different from Vícam and Pótam which showed similar values of 13.79 ± 6.03 mg/kg and 13.77 ± 3.37 (Table 1). The average values of arsenic obtained in dust samples for each village were within the limits established by Mexican legislation of 22 mg/kg [36]. However, 5.1% of the individual samples presented values above the permissible limit. On the other hand, levels of arsenic in drinking water were in the range between 7.4 and 91 μg/L, as was previously reported by Vega-Millán et al. [22]. Pótam showed significantly higher arsenic concentrations (70.0 ± 21.9 μg/L) than Vícam (23.3 ± 10.0 μg/L) and Cócorit (11.8 ± 4.4 μg/L), respectively.

### 3.3. Urinary Arsenic, MMP-9 and CC16, and Spirometric Indices for Lung Function

**Figure 2:**
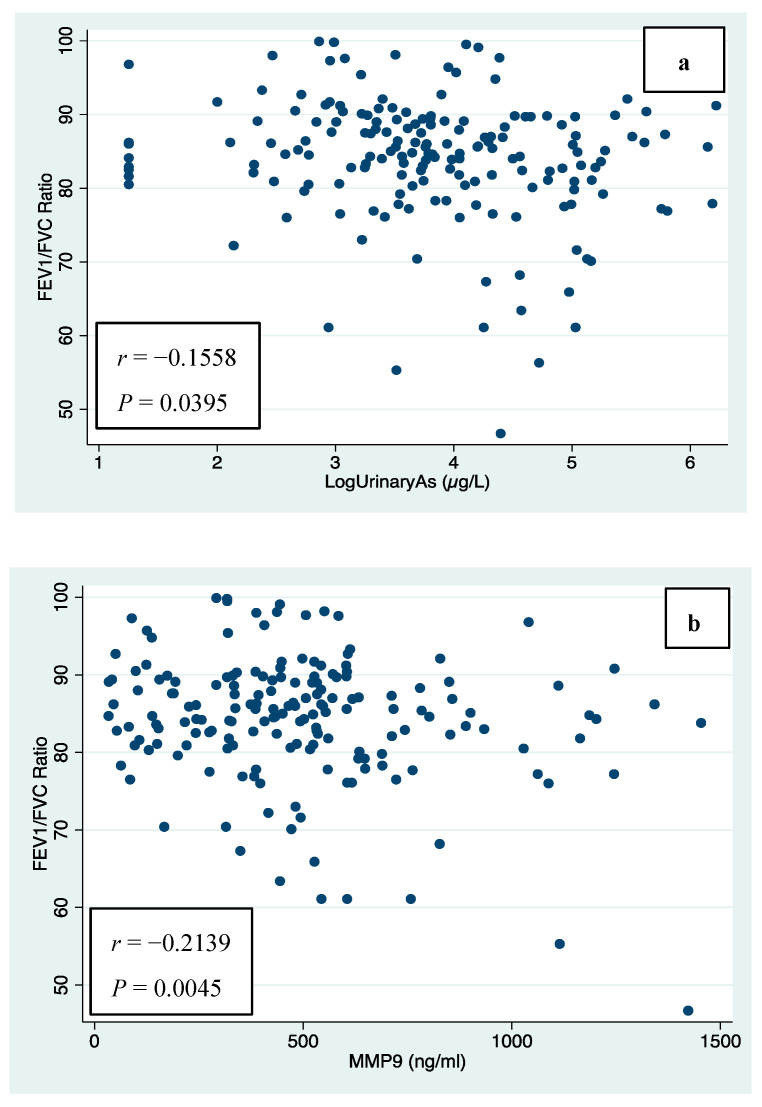
Pearson correlation between (**a**) log urinary arsenic and FEV_1_/FVC ratio (**b**) serum MMP-9 levels and FEV_1_/FVC ratio.

The levels of urinary arsenic are shown in Table 2, with a median value of 44.13 μg/L for the total participants. Children from Pótam, exposed to the highest levels of arsenic by drinking water, excreted the highest levels of arsenic in their urine with a median value of 96.70 μg/L, significantly different compared with children from Vícam and Cócorit, exposed to lower arsenic concentrations from drinking water, with median urinary arsenic levels of 26.35 μg/L and 33.75 μg/L, respectively. Urinary arsenic was significantly higher in boys with a median of 51.92 µg/L (27.1–128.3) compared with girls with a median of 41.5 µg/L (20.9–82.7). Serum MMP-9 concentrations were significantly higher in Pótam (535.16 ng/mL), compared with Vícam (437.18 ng/mL) and Cócorit (322.03 ng/mL). For serum CC16, the relation was different, with the highest concentration in Cócorit (38.39 ng/mL) and the lowest in Vícam (26.02 ng/mL), both significantly different from Pótam (29.20 ng/mL). In relation with these two biomarkers (MMP-9 and CC16), we did not find statistically significant differences by gender (*p* > 0.05). The spirometry results for children by village are shown also in Table 2. FVC median values were 2.23 L (IQR: 1.98, 2.82), 2.26 L (IQR: 1.66, 2.89) and 2.16 L (IQR: 1.65, 3.08) for Pótam, Vícam, and Cócorit, respectively, and non-significant differences were found by village (*p* = 0.6553). FEV1 median values were 1.83 (IQR: 1.62, 2.36), 1.96 (IQR: 1.56, 2.46), and 1.90 L (IQR: 1.49, 2.52) for Pótam, Vícam, and Cócorit, respectively, and were not significantly different by village (*p* = 0.8944) (Table 2). However, significant differences were found for FEV1/FVC ratio when Pótam was compared with Vícam and Cócorit (*p* = 0.0004), with the lowest percentage for Pótam with 83.03 (IQR: 77.55, 87.19), while for Vícam and Cócorit, the percentages were similar with 85.93 (IQR: 82.47, 91.16) and 85.78 (82.27, 90.04), respectively (Table 2). Girls showed significantly lower values for FVC (2.3 L) when compared with boys (2.5 L) [*p* < 0.0407], and similar results were observed for FEV1 with 1.9 and 2.1 L, for females and males, respectively, even though the difference was not statistically significant [*p* > 0.05]. The relationships between arsenic exposure (by dust, water and urine) and spirometric indices for lung function are shown in Table 3. Urinary arsenic was minimally and negatively related with FVC (*r* = −0.0031) and FEV1(*r* = −0.0475) even though the relationship was not significant (*p* > 0.05), but it was moderately and negatively associated with FEV1/FVC ratio, and this relationship was statistically significant (Figure 2a). Arsenic exposure via drinking water was negatively associated with FEV1 and FEV1/FVC, but only the latter was statistically significant (*p* = 0.0001). Arsenic exposure by dust was associated with a minimal and non-significant decrease in FVC and increase in FEV1/FVC. For lung biomarkers, serum MMP-9 levels were associated negatively with the FEV1/FVC ratio (*r* = −0.2139), and this relationship was statistically significant (Figure 2b), while CC16 was negatively and non-significantly associated with FVC and FEV1 [*p* > 0.05].

After adjusting by age, gender, BMI, and passive smoking, urinary arsenic was significantly negatively associated with FEV_1_/FVC (β = −0.022, *p* = 0.0222) and % predicted FEV_1_ (β = −0.723, *p* = 0.0152) (Table 4). The same relationship was observed between arsenic levels in drinking water and FEV_1_/FVC ratio (β = −0.027, *p* = 0.0000). Dust arsenic levels showed a negative and non-significant association with FVC (β = −0.040 *p* = 0.0885) and FEV_1_ (β = −0.012, *p* = 0.6816), but there was a positive and statistically significant association with FEV_1_/FVC ratio (β = 0.004, *p* = 0.0076). Increased serum MMP-9 levels were significantly associated with reduced FEV_1_/FVC ratio (β = −0.017, *p* = 0.0167), and an inverse behavior was shown for serum CC16 values, which were associated positively with FEV_1_/FVC ratio, although this association was non-significant (β = 0.004, *p* = 0.3120). Table S1 is included in the Supplementary Material and shows a linear association between urinary arsenic and environmental arsenic and levels of the lung biomarkers. Urinary arsenic was associated negatively (β = −0.038, *p* = 0.1805) and positively (β = 0.203, *p* = 0.2522) with serum CC16 and MMP-9 levels, respectively. However, there was a significant negative (β = −0.003, *p* = 0.0306) and positive association (β = 0.012, *p* = 0.0000) between arsenic in drinking water and serum CC16 and MMP-9 levels, respectively. Contrary to expectations, we found a positive and negative association between arsenic in dust and serum CC16 (β = 0.050, *p* = 0.000) and MMP-9 (β = −0.090, *p* = 0.0000).

Table 5 shows the levels of urinary arsenic and lung biomarkers according to their percentage predicted FEV_1_ and FVC spirometric measures. Children with % predicted FEV_1_ values < 80 showed significantly lower levels of CC16 (*p* = 0.0148) compared with children with % predicted FEV_1_ ≥ 80, with levels of 29.0 and 34.6 ng/L, respectively. Urinary arsenic and MMP-9 showed slightly higher concentrations in children with % predicted FEV_1_ < 80 with values of 45.6 μg/L and 506.4 ng/mL, respectively, compared with children with % predicted FEV_1_ ≥ 80, with values of 43.3 and 446.0 ng/mL, respectively; however, even these differences were not statistically significant [*p* > 0.05]. The children did not show statistically significant differences in urinary arsenic levels and biomarkers concentrations with % predicted FVC measures ≥80 or <80.

Table S2 is included in Supplementary Material, and it shows the spirometric pattern for our children who had percentages of 54.3, 40.0 and 5.71% for normal, restrictive, and obstructive pattern, respectively. Children with restrictive spirometric pattern had the highest arsenic concentrations in urine (55.51 μg/L); however, these were not statistically significant different compared with the other two groups.

In the study population, colds (82.9%), throat (73.1%), and ear infection (28.6%) were the most common respiratory infections or symptoms, followed by shortness of breath (18.3%) (Table S3, contained in Supplementary Materials). The difference in the frequency of colds in Pótam (highest exposure group) compared with Cócorit (lowest exposure group), was close to significant [*p* = 0.0512]. Allergies and bronchitis were the most common conditions, reported in 12.6% and 10.9% of children, respectively, and bronchitis showed significant differences between groups (*p* = 0.0383) with the highest percentages for Pótam and Vícam, the villages with the highest arsenic levels in drinking water. Asthma and pneumonia were the conditions least common in our children with percentages ≤ 5.5%.

## 4. Discussion

The results of this study identify important associations between multiple routes of arsenic exposure, biomarkers, and measures of lung function in children. This includes evaluation of exposure to arsenic in dust and drinking water. Our arsenic levels in dust were similar or higher compared with some reports in the literature. For example, Gabarrón et al. [37] studied the community of Mazarrón in Spain, located near mining wastes, and found arsenic levels in urban dust samples of 31.0 mg/kg, similar to the levels found for some dust samples from Cócori and Pótam, with 35.9 and 33.8 mg As/kg, respectively. For different areas in Pakistan, Alamdar et al. [38] reported arsenic concentrations in dust from outdoor surfaces in the range of 2.33 to 10.64 mg/kg. These latter results were three times lower than the concentrations found in some samples of our study villages. Previous Mexican studies carried out by Garcia-Rico et al. [6] reported arsenic levels similar to our results for dust samples from southern Sonora, with a range of concentrations between <10 to 27 mg/kg and 10.4 to 21.1 mg/kg for agricultural and backyards soils, respectively. In addition, an earlier study carried out in playground dust from some schools in Hermosillo, Sonora, showed arsenic levels in the range of 10.5 to 23.1 mg/kg with a mean value of 16.9 mg/kg, concentrations within the range obtained in this current study [39]. For arsenic in drinking water, the three villages exceeded the new Mexican guideline of 10 μg/L [40]. Similar results have been reported previously for this area [14].

To our knowledge, this is the first study carried out in Mexican children, which relates spirometry indices for lung function with low-level arsenic exposure from drinking water, and urinary arsenic, besides dust. In the literature, most studies which relate arsenic exposure with spirometry indicators have been carried out in adults, and most of them showed decline of FVC and FEV_1_ measurements, with increasing arsenic exposure by drinking water at levels higher than 100 μg/L, but not at lower concentrations [41,42]. However, other authors have found reduction in spirometry values at arsenic levels lower than 60 μg/L [43,44,45], as was observed in our children exposed to arsenic levels lower than 91 μg/L. In our study, girls showed significantly lower values for FVC when compared with boys, and similar results were observed for FEV_1_, even though our boys showed a more pronounced negative association with the FEV_1_/FVC ratio (β = −2.17, *p* > 0.05) compared with girls (β = −1.12, *p* > 0.05). This behavior could be explained because our boys had higher urinary arsenic levels than girls. These latter results were similar with previous data reported for children from Bangladesh [18].

In the Yaqui children, the most affected spirometry parameter was the FEV_1_/FVC ratio, which showed a significant decline with increased exposure to arsenic by urine and water. Until now, there are limited similar studies which include data for this spirometric ratio with inconsistent results. Wang et al. [46], Parvez et al. [47] and von Ehrenstein et al. [48] found a significant decrease in the (FEV_1_/FVC) ratio in adults from China, Bangladesh, and India; meanwhile, Nafees et al. [49], Parvez et al. [42], Das et al. [43], and Powers et al. [50] observed a non-significantly decreased in FEV_1_/FVC ratio with increased exposure to arsenic by urine or drinking water in adults from India, Bangladesh, Pakistan, and the US, respectively. On the other hand, Feng et al. [51] found a non-significant, but positive association between urinary arsenic and FEV_1_/FVC ratio in adults from China, opposite results compared with ours, which may be due to different study designs, populations, and exposure parameters [52]. Contrary to the expectations, the FEV_1_/FVC ratio showed a positive association with arsenic in dust (β = 0.004, *p* = 0.0076). In this latter case, it should be mentioned that one weakness of our study is that dust samples were not taken from the indoors of the participant’s households which does not represent “the most often dust exposure” or “the most real dust exposure” for the children. To date, there are no studies carried out in children which report lung function indices associated with arsenic exposure by dust. Therefore, it is crucial to continue with studies that evaluate, apart from the drinking water route, arsenic contaminated soil, because it can be resuspended, inhaled, and deposited directly in the respiratory tract [13].

Several authors have suggested that the mechanism of action of arsenic in the lungs can enhance tissue inflammation [23,53], inducing respiratory function impairment by oxidative stress or by producing fibrosis and decline in lung function [43,54,55]. These functional changes have been correlated with protein and gene expression changes as well as morphological structural changes around the airways [56]. CC16 protects the alveolar epithelium from contaminants, its mechanism relating to the care of the respiratory tract against oxidative stress and inflammation [47,57]. CC16 is a biomarker of lung epithelium integrity and is a key protecting protein, whose levels are decreased in response to inhaled toxicants [3,23,25]. Chronic inflammation of the lung damages Clara cells, resulting in reduced levels of this protein, affecting its ability to repair the epithelium damaged by pollutants [47]. In agreement with these studies, we found significant lower levels of CC16 in our children with lower % predicted FEV_1_ values, as previously have been reported by Parvez et al. [47], who detected similar behavior for adults. Ahmed et al. [18] found that increase in the levels of CC16 in children were associated with higher lung function indices and Wang et al. [46] reported a significant negative relationship between serum CC16 concentrations and lung function parameters, indicating the potential involvement of inflammation in the effects of arsenic exposure on impaired lung function. Metalloproteinases (MMPs) are the major proteases in mammals, playing a vital role in the wound healing process, the regulation of inflammatory response, and in the development of chronic and acute lung disease [58,59,60,61,62]. Some studies have shown that chronic and persistent inflammation and oxidative stress modify the airway epithelium remodeling process, thus promoting lung injury [46]. The equilibrium between MMPs such as MMP-9 and anti-proteases TIMP-1 is a critical contributor in the airway and lung remodeling process [63]. MMPs and TIMP-1 are sensitive markers of lung inflammation in humans, and increased proteinase (MMP-9)/antiprotease (TIMP-1) activity, suggesting a potential toxic mechanism for low-level arsenic exposure [26]. MMP-9 levels increased when some lung diseases are presented as chronic obstructive pulmonary disease, lung cancer, pneumonia, etc., and it is continually secreted in the airways [62,64,65]. To date, there are very scarce reports which relate MMP-9 levels, spirometry parameters, and arsenic exposure. Our study showed that increased levels of this metalloproteinase decline the FEV_1_/FVC ratio. Similar results were reported by Olivas-Calderón et al., [19] between MMP-9 levels and the spirometric indices FEV_1_ and FVC, although they did not report the FEV_1_/FVC ratio values. Wang et al. [46] reported for adults that increased sputum MMP-9 levels are associated with reduced lung function. According to Lo et al. [66], the excessive production of MMP-9 in smokers with alveolar hyperresponsiveness (AHR) had more severe deterioration of airway obstruction with reduction in predictive % FEV_1_ and % FVC compared with smokers without AHR. On the other hand, Christopoulou et al. [65] found the COPD patients are prone to produce higher levels of MMP-9 and MMP-9/TIMP-1 which is correlated with decreased % FEV_1_, compared to the control. Some studies have reported increased serum MMP-9 levels in adults and children exposed to arsenic by drinking water or dust [27,39,67]. The children in our study also showed a significant negative association between urinary arsenic and percent predicted FEV_1_, similar to results reported in previous studies [50,52,68].

The spirometric indices with abnormal patterns (obstructive or restrictive) are essential for clinical diagnosis and assessing severity of respiratory diseases, as well as to evaluate treatment and management strategies. The children from our study showed reduction in the values of FVC and especially for FEV_1_ which is an indicator of potential restrictive effects on the lung airflow. The restrictive pattern showed higher prevalence compared with the obstructive pattern (which was very low) in children with the higher urinary arsenic concentration, results in agreement with those obtained in previous studies carried out by von Ehrenstain et al. [48] in adults from India, and by Olivas- Calderón et al. [19] and Recio-Vega et al. [69] in Mexican children.

On the other hand, the children in our study did not show a significant relationship between respiratory outcomes and urinary arsenic, results in agreement with the data reported for Mexican children exposed to arsenic by drinking water [69]. This contrasted with the results reported by Parvez et al. [42], who found that arsenic in well water and urinary arsenic was related to increases in respiratory symptoms, including chronic cough and breathing difficulty, as well as significant lung function impairment. There is strong evidence that chronic exposure to arsenic by drinking water increases the susceptibility to developing respiratory disease [20,70,71,72]. A high prevalence of respiratory symptoms including chronic cough, chronic bronchitis, and shortness of breath have been reported in adults from India, Bangladesh, and Chile, exposed to high levels of arsenic [47,73,74]. Children are a vulnerable group to environmental toxicants compared with adults. In addition, they have two characteristics which magnify their exposures: their hand-to-mouth behavior and their play close to the ground [75]. Children in our study have been chronically exposed to arsenic in utero and postnatally; additionally, all of them were conceived in these villages and they have lived their whole life in the same place (early life exposure). Lantz et al. [56] reported that exposures during sensitive development time windows can irreversibly alter pulmonary structure and function in adulthood [56].

In this study, the arsenic exposure routes which had the stronger negative and statistically significant association with the spirometric indices of lung function in children were as follows: arsenic in drinking water and urine, both with low or moderate levels. Moreover, arsenic in drinking water increased the levels of serum MMP-9 and decreased serum CC16 levels in our children, increasing the potential risk of lung function damage.

There were some limitations in our research; for example, a cross-sectional design was used, which limits causal inference. This epidemiological study should be conducted to obtain longitudinal data with a larger sample size so that potential confounders can be more deeply studied and would strengthen the interpretation of findings. Although our models were adjusted for age, gender, BMI, and passive smoking, other potential confounders, such as indoor air pollution, socioeconomic status and nutritional status, were not considered, even though our indigenous children were from Yaqui communities with very similar eating habits, education, traditions, and social strata.

## 5. Conclusions

Children showed spirometric indices with abnormal patterns, with the highest prevalence for the restrictive condition. Significant decline in % predicted FEV_1_ and FEV_1_/FVC ratio with increasing levels of urinary arsenic. The same pattern was observed for the FEV_1_/FVC, when the concentrations of arsenic in drinking water and serum MMP-9 levels increased. On the other hand, lower levels of CC16 were associated with lower % predicted FEV_1_ values. The children in our study showed impairment in lung function by arsenic exposure.

## Figures and Tables

**Table 1:** Anthropometric, socio-demographic characteristics, lifestyle, and arsenic levels in dust, by studied villages ^&^.

Variable Mean, sd	Pótam (*n* = 69)	Vícam (*n* = 51)	Cócorit (*n* = 55)	Total (*n* = 175)	* *p*-Value
Age (years)	9.28, 1.71	10.25, 2.86	9.95, 2.30	9.77, 2.31	0.0556
Gender% [n]					0.9893
Girls	53.62 (37)	54.90 (28)	54.55 (30)	54.29 (95)	
Boys	46.38 (32)	45.10 (23)	45.45 (25)	45.71 (80)	
BMI (kg/m^2^)	18.11, 4.03	19.30, 4.52	19.26, 4.56	18.82, 4.36	0.2208
Residence time (Years)	9.28, 1.71	10.25, 2.86	9.95, 2.30	9.77, 2.31	0.0556
Passive smoking % (n)					0.0002 *
No	78.26 (54)	90.20 (46)	56.36 (31)	74.86 (131)	
Yes	21.74 (15)	9.80 (5)	* 43.64 (24)	25.14 (44)	
Arsenic in dust (mg/kg)	13.77, 3.37	13.79, 6.03	* 20.25, 6.74	15.81, 3.01	<0.050 *

* ANOVA, Bonferroni [*p*-Value < 0.05 (statistically significant different)]; ^&^ Studied villeges: Pótam, Vícam and Cócorit.

**Table 2:** Pneumoproteins, urinary arsenic, and spirometric indices for lung function in children exposed to environmental arsenic.

Variable Median (IQR)	Pótam (*n* = 69)	Vícam (*n* = 51)	Cócorit (*n* = 55)	Total (*n* = 175)	* *p*-Value
Urinary arsenic (µg/L)	96.70 * (59.49, 153.46)	26.35 ** (15.60, 55.75)	33.75 ** (19.45, 45.06)	44.13 (25.10, 95.54)	0.0000 ***
CC16 (ng/mL)	29.20 *** (23.21, 40.35)	26.02 * (20.50, 33.94)	38.39 ** (33.29, 53.73)	32.60 (23.78, 40.35)	0.0000 ***
MMP9 (ng/mL)	535.16 *** (407.26, 722.73)	437.18 * (331.17, 550.82)	322.03 ** (130.09, 526.69)	469.51 (317.37, 605.37)	0.0013 ***
FVC (L)	2.23 *** (1.98, 2.82)	2.26 (1.66, 2.89)	2.16 (1.65, 3.08)	2.23 (1.84, 2.89)	0.6553
FEV_1_ (L)	1.83 (1.62, 2.36)	1.96 (1.56, 2.46)	1.90 (1.49, 2.52)	1.85 (1.52, 2.43)	0.8944
FEV_1_/FVC ratio	83.03 (77.55, 87.19)	85.93 * (82.47, 91.16)	85.78 * (82.27, 90.04)	84.82 (80.77, 89.35)	0.0004 ***

* *p* ≤ 0.05 IQR. Interquartile Range; ** *p* < 0.001: Mann–Whitney test [Pótam compared with Vícam or Cócorit]; *** ANOVA, Bonferroni [*p* < 0.05].

**Table 3:** Pearson correlations between environmental arsenic, urinary arsenic, pneumoproteins and spirometric indices for lung function.

Variable	Parameter	Person Coefficient	*p*-Value
* Urinary arsenic (µg/L)	FVC (L)	−0.0031	0.9674
	%predicted FVC	0.0186	0.8071
	FEV_1_ (L)	−0.0475	0.5322
	%predictedFEV_1_	−0.0676	0.3741
	FEV_1_/FVC ratio	−0.1558	0.0395
Arsenic levels in water (ug/L)	FVC (L)	0.0642	0.3984
	%predicted FVC	0.2176	0.0038
	FEV_1_ (L)	−0.0353	0.6431
	%predicted FEV_1_	0.0311	0.6833
	FEV_1_/FVC ratio	−0.2862	0.0001
Arsenic levels in dust (mg/kg)	FVC (L)	−0.0255	0.7372
	%predicted FVC	−0.0862	0.2565
	FEV_1_ (L)	0.0301	0.6929
	%predicted FEV_1_	0.0182	0.8111
	FEV_1_/FVC ratio	0.1492	0.0488
MMP-9 (ng/mL)	FVC (L)	0.1121	0.1395
	%predicted FVC	0.2382	0.0015
	FEV_1_ (L)	0.0339	0.6560
	%predicted FEV_1_	0.0939	0.2163
	FEV_1_/FVC ratio	−0.2139	0.0045
CC16 (ng/mL)	FVC (L)	−0.1170	0.1230
	%predicted FVC	−0.0535	0.4819
	FEV_1_ (L)	−0.0918	0.2268
	%predicted FEV_1_	−0.0187	0.8059
	FEV_1_/FVC ratio	0.0797	0.2946

* Urinary arsenic log transformation.

**Table 4:** Linear association between drinking water, urinary arsenic, MMP-9, and CC16 with lung function measures in children (*n* = 175), log transformed values.

Spirometric Indices Coefficient β (95%CI) *p*-Value	Arsenic Levels in Drinking Water (µg/L)	Arsenic Levels in Dust (mg/kg)	Urinary Arsenic (µg/L)	MMP-9 (ng/mL)	CC16 (ng/mL)
FVC (L)	0.306 (0.126, 0.486) 0.0008 *	−0.040 (−0.086, 0.006) 0.0885	0.251 (−0.023, 0.526) 0.0729	0.200 (0.001, 0.398) 0.0488 *	−0.033 (−0.136, 0.070) 0.5260
% of predicted FVC	nd	nd	0.791 (−1.30, 2.88) 0.0010	0.012 (0.005, 0.019) 0.0000	−0.018 (−0.292, 0.056) 0.0006
FEV_1_(L)	0.165 (−0.067, 0.397) 0.1632	−0.012 (−0.070, 0.046) 0.6816	0.175 (−0.172, 0.523) 0.3235	0.108 (−0.145, 0.360) 0.4030	−0.001 (−0.131, 0.128) 0.9836
% of predicted FEV_1_	nd	nd	−0.723 (−2.70, 1.23) 0.0152	0.004 (−0.003, 0.011) 0.0105	−0.052 (−0.216, 0.112) 0.0161
FEV_1_/FVC ratio	−0.027 (−0.039, −0.015) 0.0000 *	0.004 (0.001, 0.007) 0.0076 *	−0.022 (−0.041, −0.003) 0.0222 *	−0.017 (−0.030, −0.003) 0.0167 *	0.004 (−0.003, 0.011) 0.3120

General linear models were adjusted for age, sex, BMI, and passive smoking. *p*-Value cut off of 0.05 for all interpretations of significance. * *p* ≤ 0.05. nd: not determined.

**Table 5:** Levels of urinary arsenic and lung biomarkers in children according to their percentage of predicted FEV_1_ and FVC indices for lung function.

Variables	Predicted Values	Percentages (*n*)	* U-arsenic (µg/L) Median, (IQR) ** *p*-Value	MMP-9 (ng/mL) Median, (IQR) ** *p*-Value	CC16 (ng/mL) Median, (IQR) ** *p*-Value
FEV_1_	≥80	60.6% (106)	43.3 (21.8, 95.5)	446.0 (256.7, 584.5)	34.6 (24.9, 44.9)
	<80	39.4% (69)	45.6 (25.8, 91.5) 0.3816	506.4 (334.2, 648.3) 0.1001	29.0 (21.3, 37.4) 0.0148
FVC	≥80	73.1% (128)	42.0 (20.23, 77.74)	481.2 (303.3, 633.4)	32.2 (23.7, 40)
	<80	26.9% (47)	43.6 (25.5, 91.3) 0.9338	428.6 (317.5, 579.7) 0.2658	33.3 (24.4, 45.9) 0.4856

* Urinary arsenic. ** Mann–Whitney test (*p* ≤ 0.05). *n* = sample size.

## Data Availability

The original contributions presented in this study are included in this article and Supplementary Material. Further inquiries can be directed to the corresponding author.

## Supplementary Materials

The following supporting information can be downloaded at: https://www.mdpi.com/article/10.3390/jox15040115/s1, Table S1: Linear associations between molecular biomarkers and urinary and environmental arsenic; Table S2: Levels of urinary arsenic and molecular lung biomarkers in children according their spirometric pattern; Table S3: Respiratory symptoms and diseases more reported for children.
