# Supplementary material for: Environmental Arsenic Exposure, Biomarkers and Lung Function in Children from Yaqui Communities in Sonora, Mexico
Source: J Xenobiot. 2025 Jul 8;15(4):115. doi: 10.3390/jox15040115 (PMC12285980; doi:10.3390/jox15040115)
Supplement: Supplementary file 1 [file jox-15-00115-s001.zip › jox-3660949-supplementary.pdf]

Supplementary data

Table S1. Linear associations between biomarkers and urinary and environmental arsenic

Parameter Coefficient (95%CI) *p*-Value	CC16 (ng/ml)	MMP9 (ng/ml)
Log urinary arsenic	-0.038<br>(-0.094, 0.018)<br>0.1805	0.203<br>(-0.145, 0.551)<br>0.2522
Arsenic levels in water (µg/L)	-0.003<br>(-0.005, -0.000)<br>0.0306 *	0.012<br>(0.007, 0.017)<br>0.0000 *
Arsenic levels in dust (mg/kg)	0.050<br>(0.031, 0.068)<br>0.0000 *	-0.090<br>(-0.126, -0.053)<br>0.0000 *

General linear models were adjusted for age, sex, BMI and passive smoking.
p- Value cut off of 0.05 for all interpretations of significance.
*$p \leq 0.05$

Table S2. Levels of urinary arsenic and molecular lung biomarkers in children according their spirometric pattern.

Variables	Normal (n=95)	Restrictive (n=70)	Obstructive (n=10)	Total (n=175)	+ *p*-Value
Spirometric pattern (percentages)	54.28 %	40.00 %	5.71 %		
Urinary arsenic (µg/l) median, IQR	42.0 (20.23, 77.74)	55.51 (29.75, 112.45)	39.2 (20.9, 96.7)	44.23 (25.1, 95.54)	0.1026
CC16 (ng/ml) median, IQR	33.66 (24.24, 40.17)	33.59 (23.21, 43.89)	25.93 (21.44, 30.75)	32.60 (23.78, 40.35)	0.9692
MMP9 (ng/ml) median, IQR	469.51 (256.71, 632.13)	449.48 (318.65, 604.75)	543.17 (436.68, 605.19)	469.51 (317.37, 605.37)	0.7677

* Mann Whitney test [Normal spirometric pattern compared with restrictive pattern]
p- Value cut off of 0.05 for all interpretations of significance.

Table S3. Respiratory symptoms and diseases more reported for children by study village.

Adverse event % [n]	Pótam (n=69)	Vícam (n=51)	Cócorit (n=55)	Total (n=175)	p-Value
Cold					0.0512
Yes	87.0 %(60)	84.3 %(43)	76.4 %(42)	82.9 %(145)	
No	13.0 %(9)	15.7 %(8)	23.6 %(13)	17.1 %(30)	
Allergies					0.7185
Yes	14.5 %(10)	7.8 %(4)	14.5 %(8)	12.6 %(22)	
No	85.5 %(59)	92.2 %(47)	85.5 %(47)	87.4 %(153)	
Bronchitis					0.0383 *
Yes	8.7 %(6)	21.6 %(11)	3.6 %(2)	10.9 %(19)	
No	91.3 %(63)	78.4 %(40)	96.4 %(53)	89.1 %(156)	
Asthma					0.4898
Yes	5.8 %(4)	3.9 %(2)	5.5 %(3)	5.1 %(9)	
No	94.2 %(65)	96.1 %(49)	94.5 %(52)	94.9 %(166)	
Pneumonia					0.2944
Yes	0.0 %(0)	2.0 %(1)	0.0 %(0)	0.6 %(1)	
No	100%(69)	98.0 %(50)	100.0 % (55)	99.4 %(174)	
Ear infection					0.2818
Yes	24.6 %(17)	37.3 %(19)	25.5 %(14)	28.6 %(50)	
No	75.4 %(52)	62.7 %(32)	74.5 %(41)	71.4 %(125)	
Throat infection					0.3137
Yes	66.7 %(46)	84.3 %(43)	70.9 %(39)	73.1 %(128)	
No	33.3 %(23)	15.7 %(8)	29.1 %(16)	26.9 %(47)	
Shortness of breath					0.1644
Yes	13.0 %(9)	29.4 %(15)	14.5 %(8)	18.3 %(32)	
No	87.0 %(60)	70.6 %(36)	85.5 %(47)	81.7 %(143)	

*$p \leq 0.05$ Fisher exact test (Pótam compared with Vícam and Cócorit)
